# Environmental and Genetic Contribution to Hypertension Prevalence: Data from an Epidemiological Survey on Sardinian Genetic Isolates

**DOI:** 10.1371/journal.pone.0059612

**Published:** 2013-03-20

**Authors:** Ginevra Biino, Gianfranco Parati, Maria Pina Concas, Mauro Adamo, Andrea Angius, Simona Vaccargiu, Mario Pirastu

**Affiliations:** 1 Institute of Population Genetics, National Research Council of Italy, Sassari, Italy; 2 Institute of Molecular Genetics, National Research Council of Italy, Pavia, Italy; 3 Department of Cardiology, S.Luca Hospital, Istituto Auxologico Italiano, Milan; Chair of Cardiology & Department of Clinical Medicine and Prevention, University of Milano-Bicocca, Milan, Italy; 4 Shardna Life Sciences, Pula, Italy; 5 Institute of Genetic and Biomedical Research, National Research Council of Italy, Monserrato, Italy; University of Oxford, United Kingdom

## Abstract

**Background and Objectives:**

Hypertension represents a major cause of cardiovascular morbidity and mortality worldwide but its prevalence has been shown to vary in different countries. The reasons for such differences are still matter of debate, the relative contributions given by environmental and genetic factors being still poorly defined. We estimated the current prevalence, distribution and determinants of hypertension in isolated Sardinian populations and also investigated the environmental and genetic contribution to hypertension prevalence taking advantage of the characteristics of such populations.

**Methods and Results:**

An epidemiological survey with cross-sectional design was carried out measuring blood pressure in 9845 inhabitants of 10 villages of Ogliastra region between 2002 and 2008. Regression analysis for assessing blood pressure determinants and variance component models for estimating heritability were performed. Overall 38.8% of this population had hypertension, its prevalence varying significantly by age, sex and among villages taking into account age and sex structure of their population. About 50% of hypertensives had prior cardiovascular disease. High blood pressure was independently associated with age, obesity related factors, heart rate, total cholesterol, alcohol consumption, low education and smoking status, all these factors contributing more in women than in men. Heritability was 27% for diastolic and 36% for systolic blood pressure, its contribution being significantly higher in men (57%) than in women (46%). Finally, the genetic correlation between systolic and diastolic blood pressure was 0.74, indicating incomplete pleiotropy.

**Conclusion:**

Genetic factors involved in the expression of blood pressure traits account for about 30% of the phenotypic variance, but seem to play a larger role in men; comorbidities and environmental factors remain of predominant importance, but seem to contribute much more in women.

## Introduction

Human hypertension represents a major cause of cardiovascular morbidity and mortality affecting one in three adults worldwide [1], [2]. Recent large scale cohort studies have confirmed that high-quality epidemiological surveys are useful for the detection and prevention of such an important clinical condition [3], [4], given that periodic surveys are central to provide the information necessary for planning hypertension prevention and control programs [5 8]. Moreover, because of the impact of uncontrolled hypertension on risk of cardiovascular mortality and morbidity worldwide, it is crucial to assess the prevalence of hypertension amongst different ethnic groups [9 12], and to determine the corresponding differences in the rate of blood pressure (BP) control by anti-hypertensive treatment [13], [14]. In fact, prevalence of hypertension has been shown to differ in different countries or geographical regions. The reasons for such differences are still matter of debate, the relative contributions given by environmental and genetic factors being still poorly defined. Indeed, evidence from family studies suggests that hypertension has a complex origin, genetic factors being suggested to account for 30% of the phenotypic variability, the remainder being explained by environmental influences [15 17].

In order to perform a more in depth investigation of the respective role of environmental and genetic factors, we assessed the prevalence of hypertension and of some of its determinants in a peculiar geographically isolated area within Sardinia (Italy), called Ogliastra, characterized by a great deal of homogeneity in life style and eating habits [18]. Ogliastra villages are characterized by a slow population growth, a high rate of endogamy, consanguinity and a low level of immigration [19], [20]. Such genetic, demographic and environmental isolation represents an ideal condition for identifying the contribution of genetic and non-genetic factors to complex pathological conditions, such as hypertension, because of the reduced background variability, and this approach has proven to be extremely cost and time effective [21 26]. Thus, aims of our study were multifold. First, we estimated the prevalence of hypertension in the population of a genetic isolate in the middle-east part of Sardinia. Second, we assessed the relationship of BP levels and occurrence of hypertension with a number of environmental risk factors and comorbidities. Finally, we estimated the heritability and genetic correlation of different BP traits in this population.

## Materials and Methods

### Ethics Statement

The research protocol of the study was approved by the Ethics Committee of the Italian Ministry of Education, University and Research. The research adheres to the tenets of the declaration of Helsinki, furthermore written informed consent was obtained from all participants.

### Population Features

A full description of the methods is provided in the Extended Methods S1, and only a brief description is provided here. The study was carried out in 10 villages of the Ogliastra region, in Sardinia (Figure S1). The examination of available genealogical records, since the XVII century, demonstrated that more than 80% of the present-day population of each village descends from less than 20 founders. In the past, although neighboring, the populations of investigated villages had few contacts between them, and very few inter-marriages with each other. Furthermore, various analyses of the Y chromosome, mitochondrial DNA and genome wide high density SNPs revealed a great deal of genetic differentiation among subpopulations within Ogliastra region [20], [27], [28].

### Study Design, Data Collection and Measurements

Study design was cross-sectional and population-based. People living in the villages were invited to take part in the study by means of public proclamations and letters sent to every resident family. Respondents underwent blood sample tests, anthropometric, heart rate (HR) and BP measurements according to the ESH guidelines [29]. They were administered a standardized interview collecting socio demographic information, living habits such as smoking, alcohol consumption and exercise, clinical and family history and medication history data. Hypertension or high BP was defined as mean clinic systolic blood pressure (SBP)≥140 mmHg, and/or mean clinic diastolic blood pressure (DBP)≥90 mmHg or by the presence of ongoing treatment for hypertension, verifying the patient information slips the participants were invited to bring with them at the interview time. Awareness of hypertension was defined as the self-report of any prior diagnosis of hypertension made by a health care professional. Control of hypertension was defined as pharmacological treatment of hypertension associated with SBP<140 mmHg and DBP<90 mmHg. Routine biochemical analyses and blood cell counts were all performed in our central laboratory in Perdasdefogu by a BT 3000 Targa Chemistry analyzer (Biotecnica Instruments, Rome, Italy) and a Coulter LH Hematology analyzer (Beckman-Coulter, Brea, CA). Fieldwork, carried out by trained personnel, took place between 2002 and 2008.

### Statistical Analysis

Data quality control and standard statistical analyses were performed using STATA 11 (StataCorp, College Station, TX). Mean kinship between participants in each of the ten villages was computed in order to avoid a potential bias due to the characteristics of isolated population of this sample. Estimates of prevalence were standardized by the direct method to the age and sex structure of the 2008 Italian resident population. Linear (for quantitative determinants) and logistic (for lifestyle and comorbidities) regressions were used to assess the relation of collected parameters with BP measures and their association with hypertension, respectively. Univariate (heritability), bivariate (genetic and environmental correlation) and sex-limitation analysis of cardiovascular phenotypes were performed on 10 extended pedigrees, using a variance-components model implemented in the software Sequential Oligogenic Linkage Analysis Routines (SOLAR v. 4.2.7) [30], [31].

## Results

### Study Sample Characteristics

Within the 10460 respondents 9845 were older than 18 years, with a mean age of 49.9 (SD = 17.9) years and a maximum age of 101 years. Male subjects accounted for 42%. Average participation rate in the study was about 80%. Age and sex distribution of the studied sample are presented in Figure S2. As far as socio-demographic characteristics like education, civil status and occupation are concerned, no substantial differences were observed among villages (data not shown). Clinical and demographic data of men and women included in this study are shown in Table 1. When examining such data across the ten villages, gender differences were confirmed, and, notably, significant differences amongst villages were also observed. (Figure S3).

**Table 1:** Characteristics of Participants in the Epidemiologic Survey in Ogliastra, Sardinia, 2002–2008.

	Men (*n* = 4,162)		Women (*n* = 5,683)		
	Mean (SD)	%	Mean (SD)	%	*P* value^a^
Age (y)	49.7 (17.6)		49.9±18		0.448
Years of schooling (y)	8.5 (3.7)		8.5±4.1		0.925
Smokers		23.6		12.9	<0.0001
Daily intake of alcohol >300 ml		62.8		24.1	<0.0001
Regular exercise		14.4		9.3	<0.0001
Prior cardiovascular disease^b^		22.9		24.6	<0.05
SBP (mmHg)^c^ *	130 (16.2)		125 (18.4)		<0.0001
DBP (mmHg)^c^	83 (10.1)		79 (10.1)		<0.0001
BMI (kg/m2)	26.5 (3.9)		25.2 (5.0)		<0.0001
Blood glucose (mg/dl)	99.1 (26.4)		92.1 (23.8)		<0.0001
Triglycerides (mg/dl)	123.9 (97.6)		94.8 (52.7)		<0.0001
Total cholesterol (mg/dl)	204.5 (39.8)		208.0 (37.8)		<0.0001
HDL cholesterol (mg/dl)	46.9 (11.9)		55.6 (12.7)		<0.0001
LDL cholesterol (mg/dl)	132.7 (36.3)		133.3 (33.9)		0.353
S-uric acid (mg/dl)	5.2 (1.3)		3.5 (1.1)		<0.0001
S-magnesium (mg/dl)	1.97 (0.18)		1.94 (0.18)		<0.0001
S-sodium (mg/dl)	137.2 (2.6)		137.0 (2.7)		0.0009
S-potassium (mg/dl)	4.18 (0.36)		4.10 (0.36)		<0.0001
S-calcium (mg/dl)	9.20 (0.46)		9.15 (0.49)		<0.0001

Abbreviations: BMI, body mass index; DBP, diastolic blood pressure; SBP, systolic blood pressure; SD, standard deviation.

* *P*<0.0001.

a *P* values refer to Pearson Chi-square test comparing frequencies of categorical variables or to t-test comparing mean values of quantitative variables in men and women.

b prior cardiovascular diseases include hypertension, TIA, stroke, myocardial infarction (ischemic heart disease), heart failure, peripheral vasculopathy or evidence of atherosclerosis.

c Test on the equality of standard deviations (variances) in men and women.

### Hypertension Prevalence, Awareness, Treatment and Control

**Figure 1:**
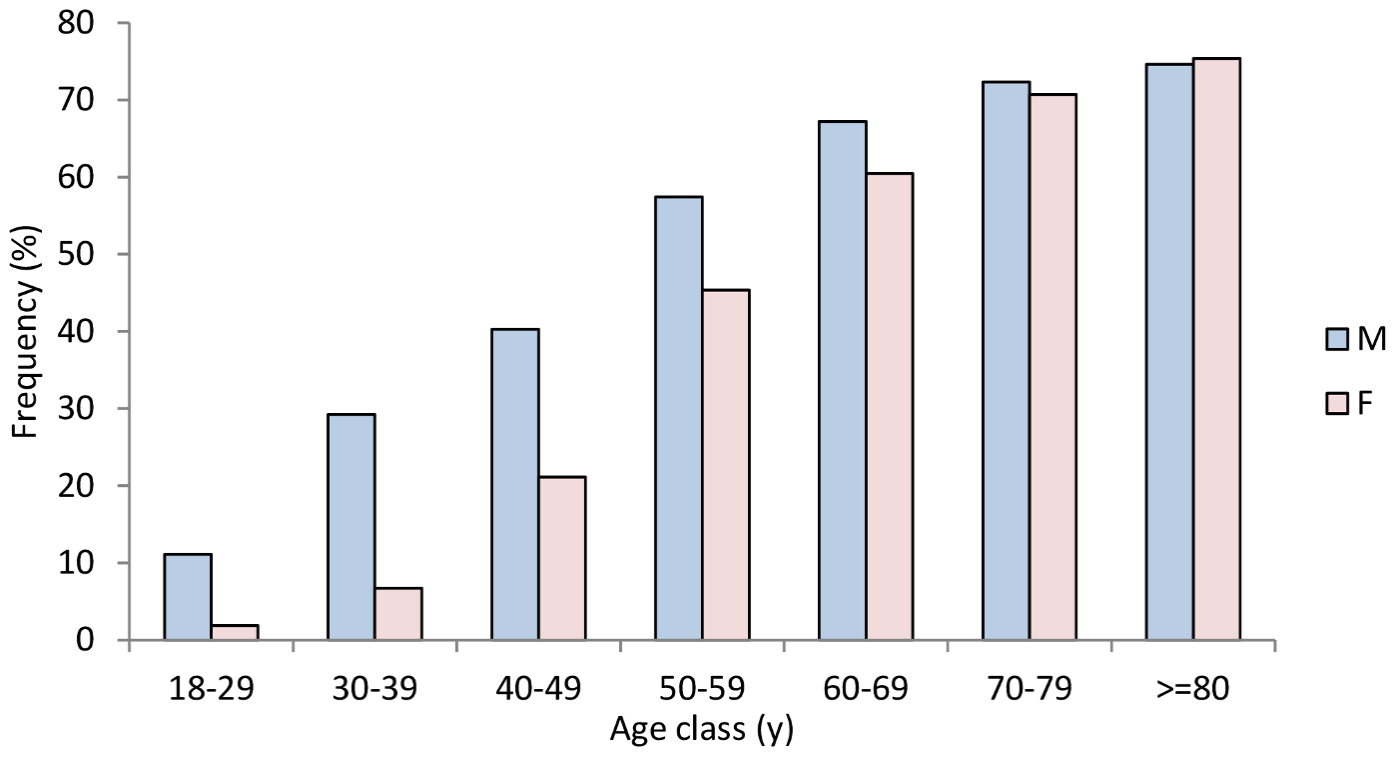
Overall age- and sex-specific prevalence of hypertension, Ogliastra, 2002–2008.

**Figure 2:**
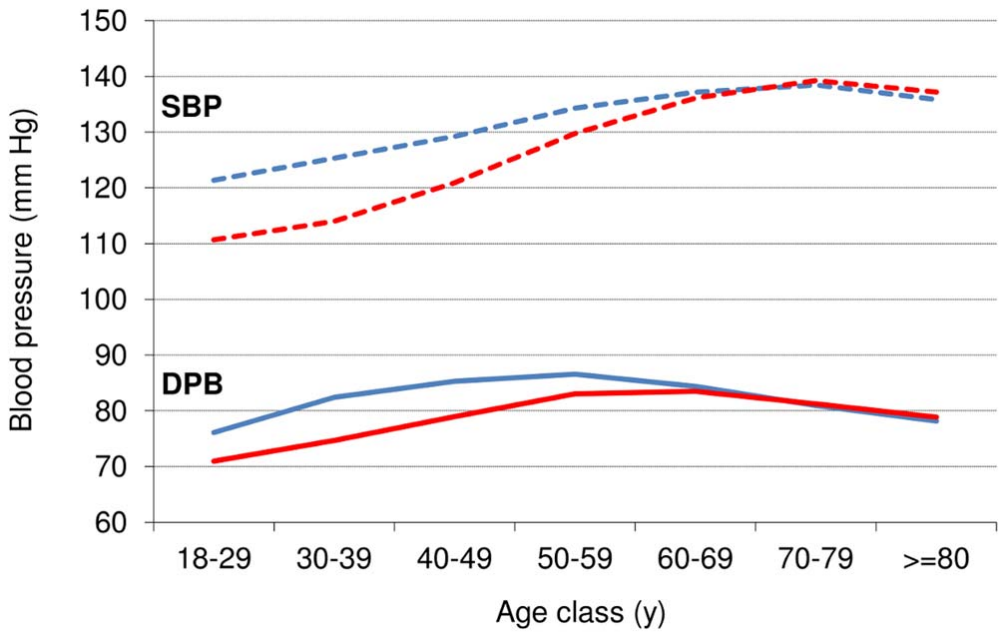
Mean systolic and diastolic blood pressure by age and sex in the overall sample, Ogliastra, 2002–2008. Men are represented by blue lines.

The overall prevalence of hypertension was 44.9% (95% CI: 43.5%, 46.2%) in men and 32.6% (95% CI: 31.6%, 33.6%) in women, ranging from 26.8% in Escalaplano to 48.5% in Loceri, with statistically significant differences amongst villages (Table 2). A significant increase in hypertension prevalence with ageing was observed in both men and women (*P*<0.001), even if the gap between genders was greater in people younger than 60 years, a pattern which is in accordance with the known trend of BP with ageing. (Figures1, 2).

**Table 2:** Prevalence^a^ of hypertension, age, BMI and alcohol consumption by village in Ogliastra, 2002–2008.

	Men	Women	Overall	Overall	Age	BMI	Alcohol consumers
	(%)	(%)	(%)	95% CI	Mean (SD)	Mean (SD)	(%)
Loceri	53.8*	43.3	48.5	45.9–51.2	50.3 (18)	26.7 (5.1)	37.8
Seulo	53.5*	40.3	47.1	44.2–50.1	49.7 (18.9)	25.5 (4.3)	40.3
Talana	50.9*	31.2	40.9	39.1–42.8	51 (19.1)	26.5 (4.9)	38.5
Baunei	48.7*	33.3	40.8	38–43.6	49 (17)	25.8 (4.2)	43.4
Ussassai	48.1*	32.8	40.7	38–43.5	52.8 (18.2)	25.2 (4.3)	40.4
Triei	46.5*	35.0	40.0	35.8–44.2	48.6 (17.5)	25.3 (4.4)	45.3
Seui	43.3*	32.2	37.9	35.4–40.4	48.8 (18)	25.1 (4.2)	43.9
Perdasdefogu	42.2*	33.2	37.6	34.6–40.6	51.6 (17)	27.2 (5.1)	37.6
Urzulei	33.0*	24.1	28.6	26–31.1	49.2 (18.2)	24.8 (4.9)	38.4
Escalaplano	29.6	24.1	26.9	24.4–29.3	50.6 (17.8)	25.5 (4.7)	35.5

Abbreviations: BMI, body mass index; CI, confidence interval; SD, standard deviation.

* *P*<0.05 for statistically significant difference in prevalence of hypertension between men and women.

a Prevalences are standardized to the age structure of the Italian resident population at 2008, using the direct method.

Overall, 54.1% of people with hypertension were aware of their condition, 45.9% were being treated and 20.4% were both treated and controlled (Table S1). Hypertensive women were generally more aware, more frequently treated and better controlled than men. Overall, half of treated subjects were on monotherapy and the most commonly used pharmaceutical classes were agents acting on the renin-angiotensin system, followed by calcium-channel blockers and beta-blocking agents (Table S2).

### Determinants of BP Levels and of Hypertension prevalence

Results of linear regression analysis of anthropometric and serological variables on BP measures are shown in Table 3. On average, increments in age, BMI, wrist circumference, waist-hip ratio, HR, and total cholesterol were associated with a BP increase, whereas an increase in levels of S-potassium was associated with a BP decrease. Looking at the partial determination coefficients, variables that explained most of SBP variance among subjects were age and BMI both in men and women, followed by wrist circumference and HR in men, and by blood glucose and total cholesterol, in women. Most important determinants of DBP were BMI and wrist circumference in both sexes, total cholesterol in men and age in women. In men only, higher levels of triglycerides were associated with a significant although slight increase in DBP, whereas higher levels of S-uric acid were associated with a significant although slight increase in SBP. Notably, for SBP the determination coefficient (adjusted R^2^) in men (20%) was about half of that found in women (40%). Results did not change substantially in a safety analysis performed by excluding subjects under antihypertensive therapy (data not shown). Finally, S-magnesium did not result significantly correlated with BP levels in the overall sample, while in the village-specific analysis some significant negative correlation between S-magnesium and BP was observed, in particular in the villages with higher levels of S-magnesium, like Seui and Urzulei (data not shown). Table 4 displays results of logistic regression analysis exploring the impact of lifestyle factors on hypertensive status. A low educational level and drinking more than half/one liter per day of wine/beer, appeared as risk factors for hypertension. Whereas smoking and doing regular exercise seemed to be protective factors.

**Table 3:** Anthropometric and Serological determinants of SBP and DBP in Ogliastra, 2002–2008.

	SBP^a^				DBP^a^			
	Men		Women		Men		Women	
	β	R^2^ (%)^b^	β	R^2^ (%)^b^	β	R^2^ (%)^b^	β	R^2^ (%)^b^
Age class (y)^c^	2.537*	9.37	4.342*	23.11	−0.079	0.12	1.103*	6.62
BMI (kg/m2)	0.516*	3.74	0.587*	6.54	0.510*	6.77	0.440*	8.38
Wrist (cm)	1.675*	2.44	0.543**	0.86	1.244*	3.08	1.138*	3.46
WHR	8.113	0.86	8.307*	1.25	10.849*	1.58	2.819	0.58
HR (bpm)	0.222*	2.04	0.191*	0.75	0.082*	0.99	0.090*	0.66
Total Chol (mg/dl)	0.030*	1.02	0.041*	2.08	0.038*	3.18	0.036*	3.20
Triglycerides (mg/dl)	−0.001	0.05	0.007	0.40	0.005**	0.77	0.002	0.17
Blood glucose (mg/dl)	0.028*	0.76	0.060*	2.30	0.003	0.07	0.007	0.33
S-potassium (mg/dl)	−2.066*	0.12	−2.648*	0.33	−1.990*	0.22	−1.584*	0.18
S-uric acid (mg/dl)	0.425**	0.52	0.386	0.70	0.072	0.14	0.131	0.32

Abbreviations: β, regression coefficient; DBP, diastolic blood pressure; SBP, systolic blood pressure.

* *P*<0.001;

** *P*<0.05.

a HDL and LDL cholesterol, S-sodium and S-calcium were excluded from the final multiple models because their coefficients were statistically significant only in the simple regression models.

b Contribution of single independent variables to the explanation of the dependent variables (partial R^2^).

c Age classes: 18–29, 30–39, 40–49, 50–59, 60–69, 70–79, >80 years.

**Table 4:** Factors associated to hypertensive status in Ogliastra, 2002–2008.

	OR^a^	95% CI	*P-*value
Education			
University	1.0		
Elementary	1.65	1.26–2.15	<0.0001
Junior high school	1.44	1.13–1.85	0.003
Smoking			
Never	1.0		
Current	0.82	0.71–0.95	0.011
Exercise			
Never	1.0		
1–2/week	0.78	0.62–0.98	0.035
>2/week	0.68	0.54–0.86	0.001
Beer			
Never	1.0		
>1 liter/day	2.14	1.56–2.95	<0.0001
Wine			
Never	1.0		
0.5 liters/day	1.29	1.05–1.58	0.008
>1liter/day	2.22	1.61–3.05	<0.0001

Abbreviations: CI, confidence interval; OR, odds ratio.

a Estimates were obtained by multiple logistic regression adjusting for age, sex and BMI.

### Comorbidities

The prevalence of metabolic syndrome in hypertensives was about five times that found in normotensives, while the prevalence of obesity was more than three times and those of diabetes and hyperuricaemia were two times greater than in normotensives (Table S3).

Prevalences of hypertension comorbidities in Ogliastra are comparable to those observed in outbred populations (Table S4), but again, statistically significant differences in the frequency of the various comorbidities were observed among villages, (Figure S4). We also investigated whether there was any relation between the distribution among villages of these hypertension-related pathologic conditions and the prevalence of hypertension. We therefore compared across villages the relative frequencies of subjects affected by hypertension only and of those in whom hypertension was combined with most prevalent comorbidities. The frequency of subjects affected by hypertension only was quite stable across villages (around 12.5%) being however significantly higher in males than in females (16% vs 9.8%, *P*<0.0001), while hypertension was variably associated with different comorbidities in different villages (Figure S5).

### Heritability

Village-specific heritability estimates for all BP traits are presented in Figure S6. Since only DBP heritability was significantly different amongst villages, we also carried out a further analysis pooling together pedigrees of all the villages. Global heritability estimates, obtained taking into account the effect of age, sex, BMI, alcohol consumption, education, smoking and exercise, are shown in Table 5.

**Table 5:** Heritability estimates of BP measures along with proportion of variance explained by covariates.

	h^2^ (%)	95% CI	Var by cov^a^ (%)
Systolic blood pressure	36.2	30.9–41.5	30.8
Diastolic blood pressure	27	22.1–31.9	21.5
Pulse pressure	29.6	24.3–34.8	19.2
Heart rate	21.3	16.1–26.5	2.6

Ogliastra, 2002–2008.

Abbreviations: CI, confidence interval; h^2^, heritability.

a The proportion of variance explained by covariates refers to the variables included in the model: sex, age, BMI, alcohol consumption, education, smoking and, exercise.

Significant differences in the dispersion (standard deviation) of SBP values came across between men and women (Table 1), with a smaller portion of SBP variance in males accounted for by the many covariates included in the model. To evaluate sex-specific effects on the variation of BP traits we used a standard sex-limitation modeling approach that allows testing for specific patterns of interaction, such as genotype by sex (G×S) interactions (Methods S1). Sex-specific heritability for SBP was 46% in women and 57% in men, with a genetic correlation between men and women significantly <1 (*P*<0.05) and with genetic variances significantly different by gender (*P*<0.05), indicating a G×S interaction.

Finally, in bivariate analyses, the genetic correlation between SBP and DBP was 0.74; it was significantly greater than zero and less than one, indicating incomplete pleiotropy (ρ_G_≠1 and ρ_G_≠0, *P*<1.0E−22). The environmental correlation (i.e. the correlation explained by environmental factors) was 0.59, significantly greater than zero (ρ_E_≠0, *P*<0.05), whereas bivariate heritability was 23%, revealing the existence of genetic influences shared between SBP and DBP values which are likely to contribute to their phenotypic correlation (ρ_P_ = 0.64).

## Discussion

This study provides for the first time information on hypertension prevalence and determinants in different Sardinian isolates, whose genetic background could be investigated in details. The interest of our results is due to the fact that Ogliastra isolated populations, thanks to their characteristic environmental homogeneity, offer quite a unique opportunity to study distribution of BP values and prevalence of hypertension controlling for exogenous factors. Moreover, given that the clinical and epidemiological features of hypertension in these peculiar isolates are comparable with those observed in outbred populations, results we obtained are likely to be applicable also to other populations. In fact, prevalence, awareness, treatment, and control of hypertension we found in Ogliastra population is similar to that of other European countries [13], [32]. Furthermore we were able to identify many of the well known risk factors for hypertension, such as age, obesity related factors, dyslipidemia, diabetes, alcohol consumption and low education [33], [34].

An additional merit of this study is related to the possibility of separately assessing prevalence of hypertension and of its comorbidities in different sub-isolates of the Ogliastra region. Interestingly, we found a disproportionate burden of high BP in some of the villages (Loceri and Seulo) whose populations have a prevalence of hypertension two times higher than in other villages (Urzulei and Escalaplano). In Loceri the higher hypertension seems to be associated to a high prevalence of obesity and metabolic syndrome, (23% and 26% respectively), while in Seulo the higher hypertension seems to be associated to a high prevalence of hypercholesterolemia (20%). Conversely in villages where hypertension was lowest (Urzulei and Escalaplano), also the respective prevalence of obesity (13% and 14%), metabolic syndrome (9% and 17%) and hypercholesterolemia (8% and 10%) were significantly lower than in other villages.

A noteworthy observation is that in most of the villages about 30% of subjects with high BP are characterized by having hypertension without comorbidities. These hypertensive-only subjects are significantly younger than the other hypertensives, and this condition is significantly more frequent in men than in women. The lack of association between this type of hypertension and other disorders suggests that it could be mainly due to genetic factors, and one would expect a higher heritability of this condition in males, as indeed found in our study. In addition, the overall determination coefficient of the SBP regression with a number of possible predictors was about 50% smaller in men than in women, as if such variables had a smaller effect in men, leaving a larger portion of the trait variability unexplained by the model and thus supporting the suggestion that genetic factors may play a larger role in males than in females.

The inverse relationship between S-magnesium and BP we found in some of the villages may be explained by the higher magnesium content of drinking water drawn from local springs nearby the villages with less hypertension. The occurrence of a relationship between a rich dietary magnesium intake and reduced BP in humans was demonstrated by many epidemiological and clinical investigations. [35], [36].

Another interesting finding of this study, in line with the results of previous epidemiologic surveys, is that smoking appeared to be inversely associated with hypertension. A likely explanation is that smokers have a lower BMI than non smokers, which might have affected such relation in spite of previous demonstration of the acute pressure effect of cigarette smoking [37]. Moreover, in our study BP was taken after a 40 minutes interview in the clinic without the possibility of smoking, so that these BP measures are no longer affected by the acute pressor effect of the last cigarette. An additional explanation of these apparent protective effects of smoking on hypertension might be related to the fact that physicians had likely advised their hypertensive patients to quit smoking, so that proportion of smoking hypertensives is lower than the proportion of not smoking ones.

Finally, our study offers further information on the heritable component of arterial BP. Cardiovascular phenotypes showed a considerable genetic component, ranging from 21% for HR to 36% for SBP, consistently with results obtained in different populations [38 41]. At variance from other studies, covariates we included in the analysis accounted for a large portion of the phenotypic variance (up to 31%), thus heritability estimates were not inflated by their effect. Genetic correlation analyses between SBP and DBP indicated that they partly share a common genetic basis but the observed incomplete pleiotropy between them suggests the existence of trait-specific sets of genes controlling each BP parameter. Along the same line, although SBP and DBP values are influenced to a great extent by the same environmental factors, they still appear to be at least in part influenced by unique environmental determinants.

As to the differences in DBP heritability estimates observed in some of the villages they may be due to the peculiarity of these populations, which represent quite distinct sub-isolates. Distinctive founder effects and genetic drift may be responsible for different frequencies in allelic variants involved in the expression of DBP. As suggested in a recent review, rare variants segregating within a few families may have much greater effect sizes accounting for the greater heritability observed in some of the villages [42].

A limitation in this study may be that only clinical and not ambulatory BP readings were taken [43]. This limitation, however, is common to most large scale epidemiologic studies, given the obvious difficulties in applying 24 h ambulatory BP monitoring in these settings. On the other side, a strength of our study is that it was conducted in a large representative sample of the Sardinian adult population using standard protocols and instruments. Furthermore, adequate training of data collectors, high subjects' response rate, performance of multiple BP measurements and, detailed information on history of hypertension and on pharmaceutical treatment ensured a high quality of this study results.

In conclusion, the analysis of data collected in this quite unique population allow us to conclude that genetic factors involved in the expression of BP traits account for about 30% of the phenotypic variance, but seem to play a larger role in men; comorbidities and environmental factors remain of predominant importance, but seem to contribute much more in women. These findings on the differential contribution of environmental and genetic factors to the prevalence of hypertension would now need to be replicated in other populations.

## Supporting Information

Figure S1 **Ogliastra region.** Geographical location of the ten villages participating in the epidemiologic survey, 2002–2008.
(TIF)

Figure S2 **Age and sex distribution of participants by village, Ogliastra, 2002–2008.**
(TIF)

Figure S3 **Mean values, along with 95% CI, of anthropometric and serum parameters amongst Ogliastra villages, 2002–2008.** Values are age- and sex adjusted by means of ANOVA. S-magnesium measurements in Escalaplano, Loceri and Triei were excluded due to technical problems of the instruments.
(TIF)

Figure S4 **Village specific prevalences of hypertension comorbidities (95% C.I.), Ogliastra, 2002–2008.** Prevalences are standardized to the age and sex structure of the Italian resident population at 2008, using the direct method. (A) Diabetes (B) Obesity (C) Metabolic Syndrome (D) Hypercholesterolemia (E) Hypomagnesemia (F) Hyperuricemia
(TIF)

Figure S5 **Distribution of hypertension comorbidities by sex and village in Ogliastra, 2002–2008.** Combinations of comorbidities represented in each graph are mutually exclusive. In each graph, columns represent frequencies of men (light blue) and women (pink) affected by a specific combination on the overall sample; prevalence of specific combinations (within hypertensives) and absolute frequency is as follows: (A) 31.6% (n = 1129), (B) 9% (n = 352), (C) 8.1% (n = 316), (D) 7% (n = 273), (E) 6.6% (n = 257), (F) 5.3% (n = 205), (G) 2.9% (n = 112), (H) 2.8% (n = 109), (I) 2.3% (n = 90), (L) 2.1% (n = 83), (M) 2.1% (n = 81), (N) 1.8% (n = 70), (O) 1.3% (n = 51), (P) 1.3% (n = 50), (Q) 1.3% (n = 51). Villages, on x axis, are: BA Baunei, ES Escalaplano, LO Loceri, PE Perdasdefogu, SI Seui, SO Seulo, TA Talana, TR Triei, UR Urzulei, US Ussassai. Obesity was defined as having a BMI ≥30; diabetes was established when subjects had fasting plasma glucose ≥126 mg/dL or current antidiabetic treatment; ATPIII (NCEP, 2001) definition was used for the diagnosis of metabolic syndrome; hypercholesterolemia was defined as having total cholesterol >250 mg/dL; hypomagnesemia as serum magnesium ≤1.8 mg/dL; whereas hyperuricemia as serum uric acid >7.0 mg/dL in men and >6.0 mg/dL in women.
(TIF)

Figure S6 **Heritability of blood pressure measures in Ogliastra villages, 2002–2008.** **Vertical bars represent 95% CI.** (A) Systolic blood pressure (B) Diastolic blood pressure (C) Heart rate (D) Pulse pressure.
(TIF)

Table S1 **Percentages of people with hypertension who are aware, treated, and controlled in the adult Ogliastra population, 2002–2008. Values are counts and %.**
(DOCX)

Table S2 **Use of anti-hypertensive treatments by villages, Ogliastra, 2002–2008.**
(DOCX)

Table S3 **Odds ratio (OR) of hypertension comorbidities, Ogliastra, 2002–2008.**
(DOCX)

Table S4 **Prevalence of hypertension comorbidities in Ogliastra, 2002–2008. Sex and age adjusted to the 2008 Italy resident population.**
(DOCX)

Methods S1 Extended Methods.
(DOCX)

## References

[1] Lawes CM, Vander Hoorn S, Rodgers A (2008) International Society of Hypertension. Global burden of blood-pressure-related disease, 2001. Lancet 371: 1513–1518. 18456100 10.1016/S0140-6736(08)60655-8

[2] The World health statistics 2012 report. World Health Organization, 2012. Accessed 6 June 2012. http://www.who.int/gho.

[3] Wolf-Maier K, Cooper RS, Banegas JR, Giampaoli S, Hense HW, et al (2003) Hypertension and blood pressure levels in 6 European countries, Canada, and the US. JAMA 289: 2363–2369. 12746359 10.1001/jama.289.18.2363

[4] Estoppey D, Paccaud F, Vollenweider P, Marques-Vidal P (2011) Trends in self-reported prevalence and management of hypertension, hypercholesterolemia and diabetes in Swiss adults, 1997–2007. BMC Public Health 11: 114. 21332996 10.1186/1471-2458-11-114 PMC3051907

[5] Hyman DJ, Pavlik VN (2001) Characteristics of patients with uncontrolled hypertension in the United States. N Engl J Med 345: 479–486. 11519501 10.1056/NEJMoa010273

[6] Lloyd-Jones DM, Evans JC, Larson MG, Levy D (2002) Treatment and control of hypertension in the community: a prospective analysis. Hypertension 40: 640–646. 12411456 10.1161/01.hyp.0000035855.44620.da

[7] Wong ND, Thakral G, Franklinn SS, L'Italien GJ, Jacobs MJ, et al (2003) Preventing heart disease by controlling hypertension: impact of hypertensive subtype, stage, age and sex. Am Heart J 145: 888–895. 12766749 10.1016/S0002-8703(02)94787-3

[8] Hajjar I, Kotchen TA (2003) Trends in prevalence, awareness, treatment, and control of hypertension in the United States, 1988–2000. JAMA 290: 199–206. 12851274 10.1001/jama.290.2.199

[9] Mathenge W, Foster A, Kuper H (2010) Urbanization, ethnicity and cardiovascular risk in a population in transition in Nakuru, Kenya: a population-based survey. BMC Public Health 10: 569. 20860807 10.1186/1471-2458-10-569 PMC2956724

[10] Esteghamati A, Meysamie A, Khalilzadeh O, Rashidi A, Haghazali M, et al (2009) Third national surveillance of risk factors of non-communicable diseases (SuRFNCD-2007) in Iran: methods and results on prevalence of diabetes, hypertension, obesity, central obesity, and dyslipidemia. BMC Public Health 9: 167. 19480675 10.1186/1471-2458-9-167 PMC2697989

[11] Bosu WK (2010) Epidemic of hypertension in Ghana: a systematic review. BMC Public Health 10: 418. 20626917 10.1186/1471-2458-10-418 PMC2910685

[12] Bharucha NE, Kuruvilla T (2003) Hypertension in the Parsi community of Bombay: a study on prevalence, awareness and compliance to treatment. BMC Public Health 3: 1. 12513697 10.1186/1471-2458-3-1 PMC140316

[13] Wolf-Maier K, Cooper RS, Kramer H, Banegas JR, Giampaoli S, et al (2004) Hypertension treatment and control in five European countries, Canada, and the United States. Hypertension 43: 10–17. 14638619 10.1161/01.HYP.0000103630.72812.10

[14] Campbell NR (2002) The Canadian Hypertension Recommendations Working Group. The 2001 Canadian Hypertension Recommendations: what is new and what is old but still important. Can J Cardiol 18: 591–603. 12107418

[15] Mein CA, Caulfield MJ, Dobson RJ, Munroe PB (2004) Genetics of essential hypertension. Hum Mol Genet 13: R169–R175. 14764624 10.1093/hmg/ddh078

[16] Dominiczak AF, Brain N, Charchar F, McBride M, Hanlon N, et al (2004) Genetics of hypertension: lessons learnt from mendelian and polygenic syndromes. Clin Exp Hypertens 26: 611–620. 15702615 10.1081/ceh-200031939

[17] Yagil Y, Yagil C (2005) The search for the genetic basis of hypertension. Curr Opin Nephrol Hypertens 14: 141–147. 15687840 10.1097/00041552-200503000-00009

[18] Cappello N, Rendine S, Griffo R, Mameli GE, Succa V, et al (1996) Genetic analysis of Sardinia: I. data on 12 polymorphisms in 21 linguistic domains. Ann Hum Genet 60: 125–141. 8839127 10.1111/j.1469-1809.1996.tb01183.x

[19] Angius A, Melis PM, Morelli L, Petretto E, Casu G, et al (2001) Archival, demographic and genetic studies define a Sardinian sub-isolate as a suitable model for mapping complex traits. Hum Genet 109: 198–209. 11511926 10.1007/s004390100557

[20] Fraumene C, Petretto E, Angius A, Pirastu M (2003) Striking differentiation of sub-populations within a genetically homogeneous isolate (Ogliastra) in Sardinia as revealed by mtDNA analysis. Hum Genet 114: 1–10. 13680359 10.1007/s00439-003-1008-3

[21] Angius A, Petretto E, Maestrale GB, Forabosco P, Casu G, et al (2002) A new essential hypertension susceptibility locus on chromosome 2p24–p25, detected by genomewide search. Am J Hum Genet 71: 893–905. 12228842 10.1086/342929 PMC378544

[22] Gianfrancesco F, Esposito T, Ombra MN, Forabosco P, Maninchedda G, et al (2003) Identification of a novel gene and a common variant associated with uric acid nephrolithiasis in a Sardinian genetic isolate. Am J Hum Genet 72: 1479–1491. 12740763 10.1086/375628 PMC1180308

[23] Prodi DA, Drayna D, Forabosco P, Palmas MA, Maestrale GB, et al (2004) Bitter taste study in a Sardinian genetic isolate supports the association of phenylthiocarbamide sensitivity to the TAS2R38 bitter receptor gene. Chem Senses 29: 697–702. 15466815 10.1093/chemse/bjh074

[24] Falchi M, Forabosco P, Mocci E, Borlino CC, Picciau A, et al (2004) Genome-wide search using an Original Pairwise Sampling Approach for Large Genealogies Identifies a New Locus for Total and LDL-cholesterol in two Genetically Differentiated, Isolates of Sardinia. Am J Hum Genet 75: 1015–1031. 15478097 10.1086/426155 PMC1182138

[25] Biino G, Palmas MA, Corona C, Prodi D, Fanciulli M, et al (2005) Ocular refraction: heritability and genome-wide search for eye morphometry traits in an isolated Sardinian population. Hum Genet 116: 152–159. 15611866 10.1007/s00439-004-1231-6

[26] Mocci E, Concas MP, Fanciulli M, Pirastu N, Adamo M, et al (2009) Microsatellites and SNPs linkage analysis in a Sardinian genetic isolate confirms several essential hypertension loci previously identified in different populations. BMC Med Genet 10: 81. 19715579 10.1186/1471-2350-10-81 PMC2741446

[27] Fraumene C, Belle EM, Castrì L, Sanna S, Mancosu G, et al (2006) High resolution analysis and phylogenetic network construction using complete mtDNA sequences in sardinian genetic isolates. Mol Biol Evol 23: 2101–2111. 16901986 10.1093/molbev/msl084

[28] Pistis G, Piras I, Pirastu N, Persico I, Sassu A, et al (2009) High differentiation among eight villages in a secluded area of Sardinia revealed by genome-wide high density SNPs analysis. PLoS One 4: e4654. 19247500 10.1371/journal.pone.0004654 PMC2646134

[29] European Society of Hypertension–European Society of Cardiology guidelines for the management of arterial hypertension (2003) Journal of Hypertension. 21: 1011–1053. 10.1097/00004872-200306000-00001 12777938

[30] Almasy L, Blangero J (1998) Multipoint quantitative-trait linkage analysis in general pedigrees. Am J Hum Gen 62: 1198–1211. 10.1086/301844 PMC1377101 9545414

[31] Martin LJ, Cole SA, Hixson JE, Mahaney MC, Czerwinski SA, et al (2002) Genotype by smoking interaction for leptin levels in the San Antonio Family Heart Study. Genet Epidemiol 22: 105–115. 11788957 10.1002/gepi.0135

[32] Wagner A, Sadoun A, Dallongeville J, Ferrières J, Amouyel P, et al (2011) High blood pressure prevalence and control in a middle-aged French population and their associated factors: the MONA LISA study. J Hypertens 29: 43–50. 20852444 10.1097/HJH.0b013e32833f9c4d

[33] Risk factors for developing high blood pressure, also called hypertension. http://www.heart.org/HEARTORG/Conditions/HighBloodPressure/UnderstandYourRiskforHighBloodPressure/Understand-Your-Risk-for-High-Blood-Pressure_UCM_002052_Article.jsp. Accessed March 6, 2012.

[34] Volpe M, Tocci G, Trimarco B, Rosei EA, Borghi C, et al (2007) Blood pressure control in Italy: results of recent surveys on hypertension. J Hypertens 25: 1491–1498. 17563573 10.1097/HJH.0b013e3280fa83a6

[35] Ascherio A, Hennekens C, Willett WC, Sacks F, Rosner B, et al (1996) Prospective study of nutritional factors, blood pressure, and hypertension among US women. Hypertension 27: 1065–1072. 8621198 10.1161/01.hyp.27.5.1065

[36] Geleijnse JM, Witteman JC, den Breeijen JH, Hofman A, de Jong PT, et al (1996) Dietary electrolyte intake and blood pressure in older subjects: the Rotterdam Study. J Hypertension 14: 737–741. 10.1097/00004872-199606000-00009 8793696

[37] Groppelli A, Omboni S, Parati G, Mancia G (1990) Blood pressure and heart rate response to repeated smoking before and after beta-blockade and selective alpha 1 inhibition. J Hypertens Suppl 8: S35–40. 1981075

[38] Brown WM, Beck SR, Lange EM, Davis CC, Kay CM, et al.. (2003) Age-stratified heritability estimation in the Framingham Heart Study families. BMC Genet Suppl 1: S32. 10.1186/1471-2156-4-S1-S32 PMC1866468 14975100

[39] Bochud M, Bovet P, Elston RC, Paccaud F, Falconnet C, et al (2005) High heritability of ambulatory blood pressure in families of East African descent. Hypertension 45: 445–450. 15699448 10.1161/01.HYP.0000156538.59873.86

[40] Pilia G, Chen W-M, Scuteri A, Orrú M, Albai G, et al (2006) Heritability of Cardiovascular and Personality Traits in 6,148 Sardinians. PLoS Genet 2: e132. 16934002 10.1371/journal.pgen.0020132 PMC1557782

[41] van Rijn MJ, Schut AF, Aulchenko YS, Deinum J, Sayed-Tabatabaei FA, et al (2007) Heritability of blood pressure traits and the genetic contribution to blood pressure variance explained by four blood-pressure-related genes. J Hypertens 25: 565–570. 17278972 10.1097/HJH.0b013e32801449fb

[42] Doris PA (2011) The genetics of blood pressure and hypertension: the role of rare variation. Cardiovasc Ther 29: 37–45. 21129164 10.1111/j.1755-5922.2010.00246.x PMC3562708

[43] Mancia G, Di Rienzo M, Parati G (1993) Ambulatory blood pressure monitoring use in hypertension research and clinical practice. Hypertension 21: 510–524. 8458650 10.1161/01.hyp.21.4.510

