# Supplementary figures and images for: Environmental and Genetic Contribution to Hypertension Prevalence: Data from an Epidemiological Survey on Sardinian Genetic Isolates
Source: PLoS One. 2013 Mar 20;8(3):e59612. doi: 10.1371/journal.pone.0059612 (PMC3603911; doi:10.1371/journal.pone.0059612)

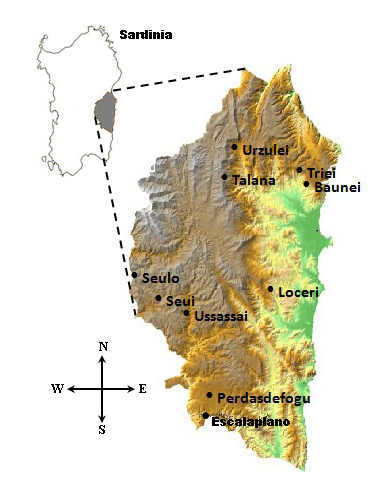

Supplement: Figure S1 — Ogliastra region. Geographical location of the ten villages participating in the epidemiologic survey, 2002–2008. (TIF) [file pone.0059612.s001.tif]

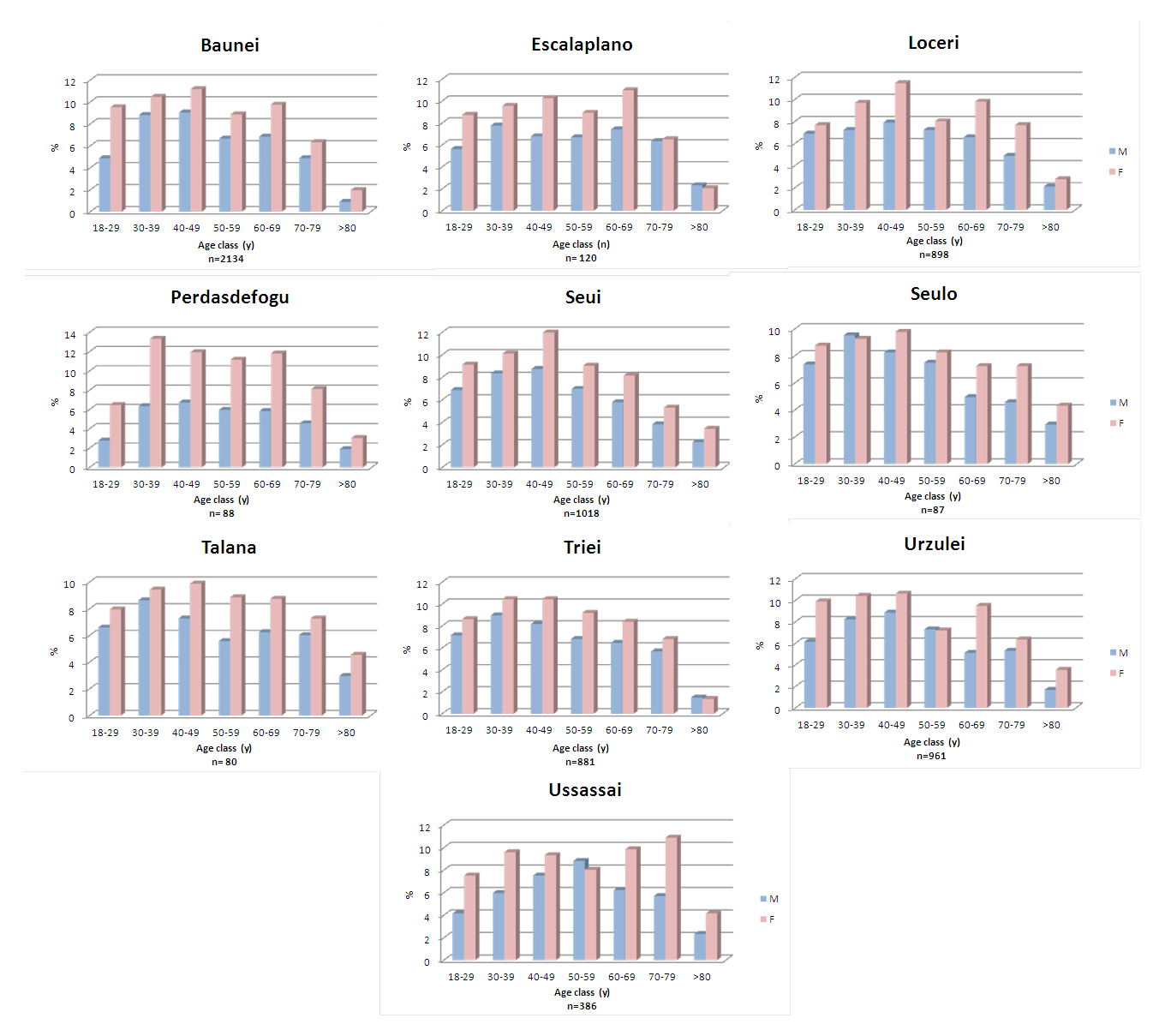

Supplement: Figure S2 — Age and sex distribution of participants by village, Ogliastra, 2002–2008. (TIF) [file pone.0059612.s002.tif]

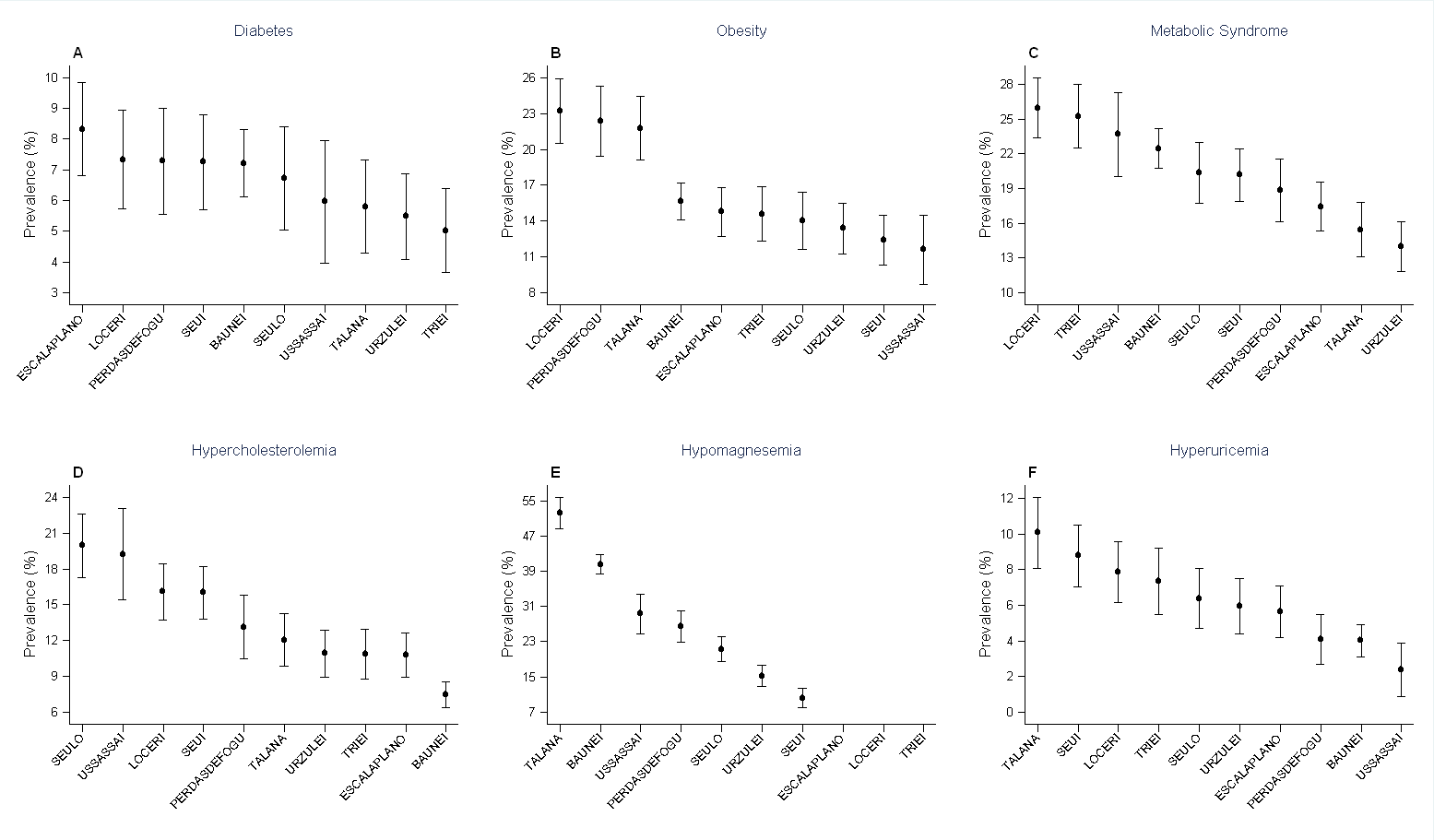

Supplement: Figure S4 — Village specific prevalences of hypertension comorbidities (95% C.I.), Ogliastra, 2002–2008. Prevalences are standardized to the age and sex structure of the Italian resident population at 2008, using the direct method. (A) Diabetes (B) Obesity (C) Metabolic Syndrome (D) Hypercholesterolemia (E) Hypomagnesemia (F) Hyperuricemia (TIF) [file pone.0059612.s004.tif]

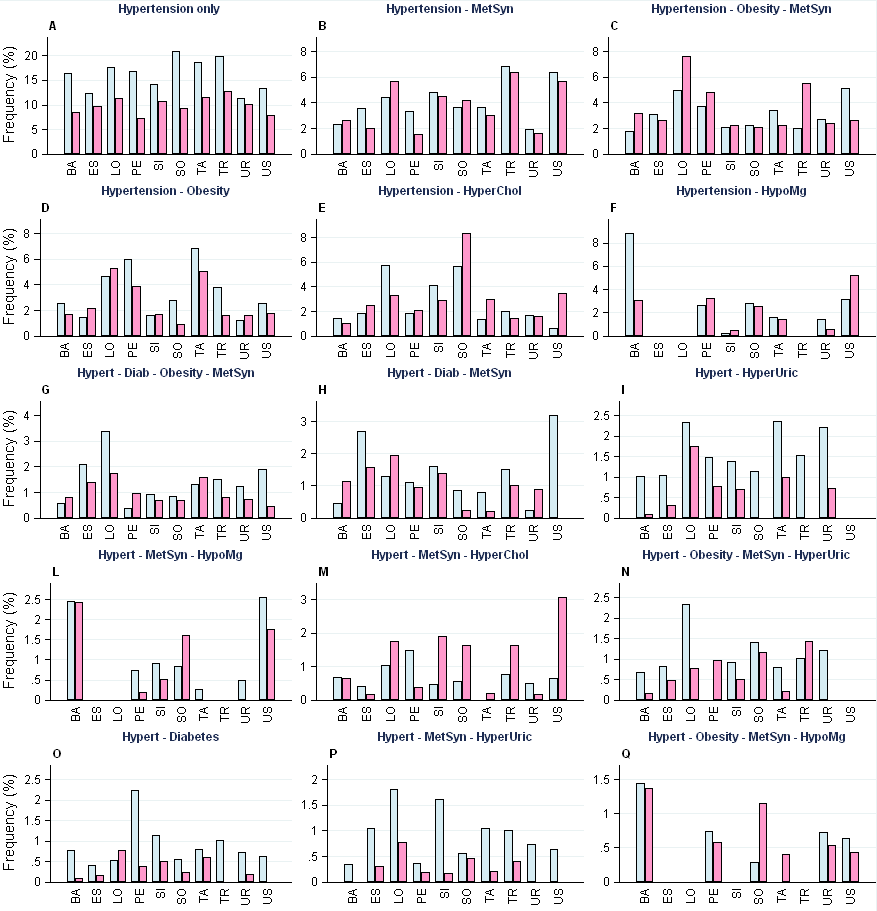

Supplement: Figure S5 — Distribution of hypertension comorbidities by sex and village in Ogliastra, 2002–2008. Combinations of comorbidities represented in each graph are mutually exclusive. In each graph, columns represent frequencies of men (light blue) and women (pink) affected by a specific combination on the overall sample; prevalence of specific combinations (within hypertensives) and absolute frequency is as follows: (A) 31.6% (n = 1129), (B) 9% (n = 352), (C) 8.1% (n = 316), (D) 7% (n = 273), (E) 6.6% (n = 257), (F) 5.3% (n = 205), (G) 2.9% (n = 112), (H) 2.8% (n = 109), (I) 2.3% (n = 90), (L) 2.1% (n = 83), (M) 2.1% (n = 81), (N) 1.8% (n = 70), (O) 1.3% (n = 51), (P) 1.3% (n = 50), (Q) 1.3% (n = 51). Villages, on x axis, are: BA Baunei, ES Escalaplano, LO Loceri, PE Perdasdefogu, SI Seui, SO Seulo, TA Talana, TR Triei, UR Urzulei, US Ussassai. Obesity was defined as having a BMI ≥30; diabetes was established when subjects had fasting plasma glucose ≥126 mg/dL or current antidiabetic treatment; ATPIII (NCEP, 2001) definition was used for the diagnosis of metabolic syndrome; hypercholesterolemia was defined as having total cholesterol >250 mg/dL; hypomagnesemia as serum magnesium ≤1.8 mg/dL; whereas hyperuricemia as serum uric acid >7.0 mg/dL in men and >6.0 mg/dL in women. (TIF) [file pone.0059612.s005.tif]

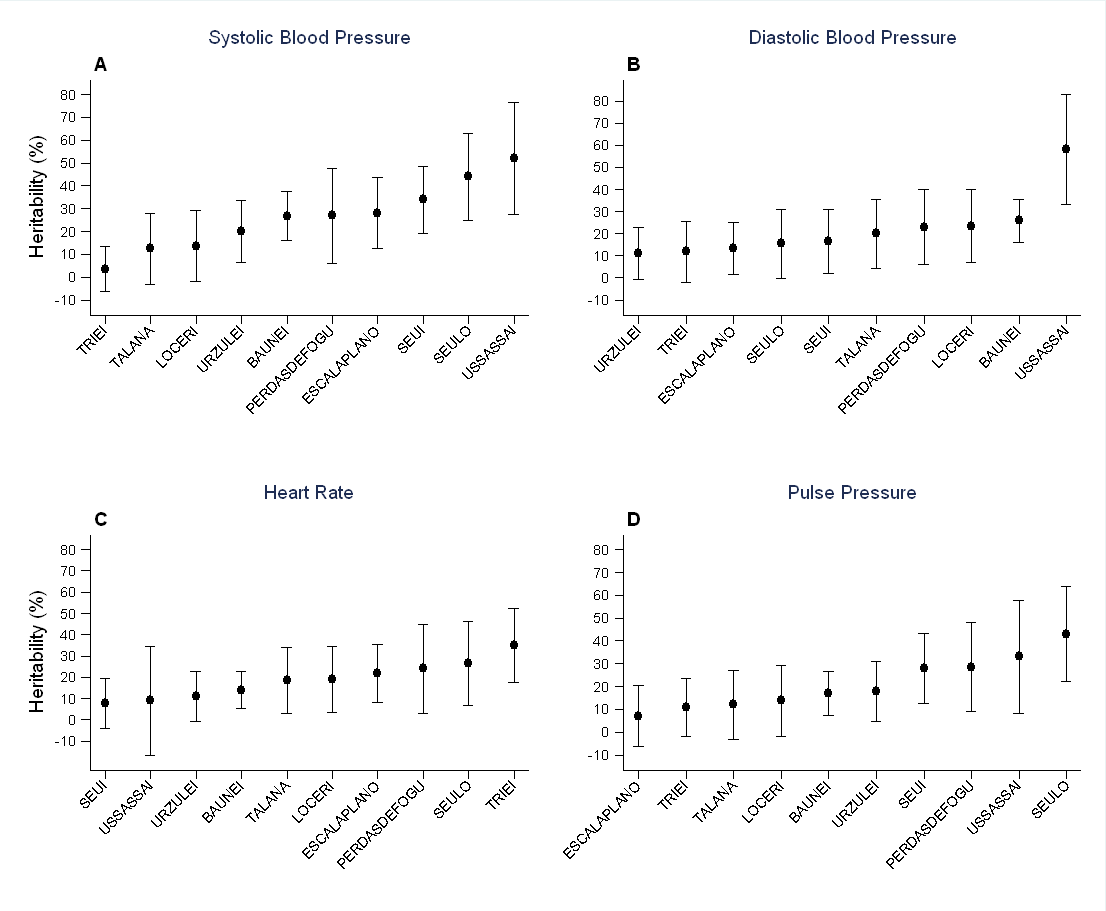

Supplement: Figure S6 — Heritability of blood pressure measures in Ogliastra villages, 2002–2008. Vertical bars represent 95% CI. (A) Systolic blood pressure (B) Diastolic blood pressure (C) Heart rate (D) Pulse pressure. (TIF) [file pone.0059612.s006.tif]
