# Supplementary material for: Environmental and Genetic Contribution to Hypertension Prevalence: Data from an Epidemiological Survey on Sardinian Genetic Isolates
Source: PLoS One. 2013 Mar 20;8(3):e59612. doi: 10.1371/journal.pone.0059612 (PMC3603911; doi:10.1371/journal.pone.0059612)
Supplement: Table S1 — Percentages of people with hypertension who are aware, treated, and controlled in the adult Ogliastra population, 2002–2008. Values are counts and %. (DOCX) [file pone.0059612.s007.docx]

**Table S1.** Percentages of people with hypertension who are aware, treated, and controlled in the adult Ogliastra population, 2002-2008. Values are counts and %.

|  | **Hypertensives** | | | | | | | |
| --- | --- | --- | --- | --- | --- | --- | --- | --- |
|  | **Aware** | |  | **Treated** | |  | **Treated controlled*** | |
|  | **N** | **%** |  | **N** | **%** |  | **N** | **%** |
| **Overall** | 2104 | 54.1 |  | 1783 | 45.9 |  | 791 | 20.4 |
| **Men** | 898 | 46.6 |  | 738 | 38.3 |  | 345 | 17.9 |
| **Women** | 1206 | 61.6 |  | 1045 | 53.4 |  | 446 | 22.8 |
| **Baunei** | 377 | 43.63 |  | 315 | 36.5 |  | 133 | 15.4 |
| **Escalaplano** | 204 | 63.95 |  | 172 | 53.9 |  | 107 | 33.5 |
| **Loceri** | 242 | 52.84 |  | 203 | 44.3 |  | 69 | 15.1 |
| **Perdasdefogu** | 206 | 64.98 |  | 187 | 59 |  | 73 | 23 |
| **Seui** | 214 | 56.76 |  | 185 | 49.1 |  | 84 | 22.3 |
| **Seulo** | 194 | 51.6 |  | 158 | 42 |  | 60 | 16 |
| **Talana** | 236 | 62.93 |  | 204 | 54.4 |  | 80 | 21.3 |
| **Triei** | 173 | 48.6 |  | 156 | 43.8 |  | 75 | 21.1 |
| **Urzulei** | 175 | 64.81 |  | 133 | 49.3 |  | 82 | 30.4 |
| **Ussassai** | 83 | 47.7 |  | 70 | 40.2 |  | 28 | 16.1 |

* Proportion of hypertensives on an antihypertensive medication with SBP & DBP < 140/90 mmHg
