# Supplementary material for: Environmental and Genetic Contribution to Hypertension Prevalence: Data from an Epidemiological Survey on Sardinian Genetic Isolates
Source: PLoS One. 2013 Mar 20;8(3):e59612. doi: 10.1371/journal.pone.0059612 (PMC3603911; doi:10.1371/journal.pone.0059612)
Supplement: Table S2 — Use of anti-hypertensive treatments by villages, Ogliastra, 2002–2008. (DOCX) [file pone.0059612.s008.docx]

**Table S2.** Use of anti-hypertensive treatments by villages, Ogliastra, 2002-2008

|  | **Angiotensin inhibitors** | |  | **αAdrenergic blockers** | |  | **β-blockers** | |  | **Calcium-channel blockers** | |  | **Diuretics** | |  | **Cardiac therapy** | |
| --- | --- | --- | --- | --- | --- | --- | --- | --- | --- | --- | --- | --- | --- | --- | --- | --- | --- |
|  | **N** | **%** |  | **N** | **%** |  | **N** | **%** |  | **N** | **%** |  | **N** | **%** |  | **N** | **%** |
| **Overall** | 1144 | 44.64 |  | 107 | 4.17 |  | 424 | 16.54 |  | 442 | 17.25 |  | 296 | 11.55 |  | 150 | 5.85 |
| **Baunei** | 206 | 46.29 |  | 12 | 2.70 |  | 92 | 20.67 |  | 73 | 16.40 |  | 38 | 8.54 |  | 24 | 5.39 |
| **Escalaplano** | 132 | 53.44 |  | 14 | 5.67 |  | 44 | 17.81 |  | 30 | 12.15 |  | 18 | 7.29 |  | 9 | 3.64 |
| **Loceri** | 112 | 38.49 |  | 10 | 3.44 |  | 41 | 14.09 |  | 67 | 23.02 |  | 47 | 16.15 |  | 14 | 4.81 |
| **Perdasdefogu** | 129 | 43.73 |  | 23 | 7.80 |  | 37 | 12.54 |  | 51 | 17.29 |  | 37 | 12.54 |  | 18 | 6.10 |
| **Seui** | 116 | 44.62 |  | 11 | 4.23 |  | 33 | 12.69 |  | 37 | 14.23 |  | 41 | 15.77 |  | 22 | 8.46 |
| **Seulo** | 103 | 44.59 |  | 12 | 5.19 |  | 31 | 13.42 |  | 45 | 19.48 |  | 28 | 12.12 |  | 12 | 5.19 |
| **Talana** | 129 | 42.30 |  | 10 | 3.28 |  | 47 | 15.41 |  | 62 | 20.33 |  | 38 | 12.46 |  | 19 | 6.23 |
| **Triei** | 92 | 42.99 |  | 8 | 3.74 |  | 49 | 22.90 |  | 36 | 16.82 |  | 14 | 6.54 |  | 15 | 7.01 |
| **Urzulei** | 84 | 49.12 |  | 4 | 2.34 |  | 25 | 14.62 |  | 25 | 14.62 |  | 23 | 13.45 |  | 10 | 5.85 |
| **Ussassai** | 41 | 39.42 |  | 3 | 2.88 |  | 25 | 24.04 |  | 16 | 15.38 |  | 12 | 11.54 |  | 7 | 6.73 |
