# Supplementary material for: Environmental and Genetic Contribution to Hypertension Prevalence: Data from an Epidemiological Survey on Sardinian Genetic Isolates
Source: PLoS One. 2013 Mar 20;8(3):e59612. doi: 10.1371/journal.pone.0059612 (PMC3603911; doi:10.1371/journal.pone.0059612)
Supplement: Table S3 — Odds ratio (OR) of hypertension comorbidities, Ogliastra, 2002–2008. (DOCX) [file pone.0059612.s009.docx]

**Table S3.** Odds ratio (OR) of hypertension comorbidities, Ogliastra, 2002-2008.

|  | **OR** | **95% CI** | ***P*** |
| --- | --- | --- | --- |
| **Diabetes** | 1.9 | 1.6 – 2.3 | <0.0001 |
| **Obesity** | 3.3 | 2.9 – 3.7 | <0.0001 |
| **Metabolic syndrome** | 4.8 | 4.2 – 5.5 | <0.0001 |
| **Hypercholesterolemia** | 1.4 | 1.2 – 1.6 | <0.0001 |
| **Hypomagnesemia** | 1.1 | 0.9 – 1.3 | 0.083 |
| **Hyperuricemia** | 2.2 | 1.8 – 2.7 | <0.0001 |

Estimates were obtained from logistic regression models with the comorbidity as dependent variable and hypertension as independent variabile, with normotensive status as reference category (OR=1) and adjusting for age and gender.
