# Supplementary material for: Environmental and Genetic Contribution to Hypertension Prevalence: Data from an Epidemiological Survey on Sardinian Genetic Isolates
Source: PLoS One. 2013 Mar 20;8(3):e59612. doi: 10.1371/journal.pone.0059612 (PMC3603911; doi:10.1371/journal.pone.0059612)
Supplement: Table S4 — Prevalence of hypertension comorbidities in Ogliastra, 2002–2008. Sex and age adjusted to the 2008 Italy resident population. (DOCX) [file pone.0059612.s010.docx]

**Table S4.** Prevalence of hypertension comorbidities in Ogliastra, 2002-2008. Sex and age adjusted to the 2008 Italy resident population.

|  |  | **Overall** | |  | **Men** | |  | **Women** | |
| --- | --- | --- | --- | --- | --- | --- | --- | --- | --- |
|  |  | **%** | **95% CI** |  | **%** | **95% CI** |  | **%** | **95% CI** |
| **Diabetes** |  | 6.9 | 6.4-7.4 |  | 8.1 | 7.3-8.9 |  | 5.8 | 5.3-6.4 |
| **Obesity** |  | 16.4 | 15.7-17.1 |  | 16.6 | 15.5-17.7 |  | 15.9 | 15-16.8 |
| **Metabolic syndrome** |  | 19.7 | 18.9-20.4 |  | 19.7 | 18.6-20.9 |  | 19.1 | 18.1-20 |
| **Hypercholesterolemia** |  | 12.1 | 11.4-12.7 |  | 11.9 | 10.9-12.9 |  | 11.9 | 11.1-12.8 |
| **Hypomagnesemia** |  | 28 | 26.8-29.2 |  | 25.6 | 23.8-27.4 |  | 30.4 | 28.9-32 |
| **Hyperuricemia** |  | 6.9 | 6.4-7.5 |  | 10.4 | 9.5-11.4 |  | 3.7 | 3.2-4.1 |

Diabetes was established when subjects had fasting plasma glucose ≥ 126 mg/dL or current antidiabetic treatment; obesity was defined as having a BMI ≥ 30; ATPIII (NCEP, 2001) definition was used for the diagnosis of metabolic syndrome; hypercholesterolemia was defined as having total cholesterol>250 mg/dL; hypomagnesemia as serum magnesium ≤1.8 mg/dL; whereas hyperuricemia as serum uric acid >7.0 mg/dL in men and > 6.0 mg/dL in women.
