# Supplementary material for: Environmental and Genetic Contribution to Hypertension Prevalence: Data from an Epidemiological Survey on Sardinian Genetic Isolates
Source: PLoS One. 2013 Mar 20;8(3):e59612. doi: 10.1371/journal.pone.0059612 (PMC3603911; doi:10.1371/journal.pone.0059612)
Supplement: Methods S1 — Extended Methods. (DOCX) [file pone.0059612.s011.docx]

**EXTENDED METHODS**

***Population features***

The study was carried out in 10 villages of the Ogliastra region, in Sardinia (Figure S1). The examination of available genealogical records, since the XVII century, in combination to MtDNA and Y chromosome studies, demonstrated that more than 80% of the present-day population of each village descends from less than 20 paternal and maternal lineages. In the past, although neighboring, the populations of investigated villages had few contacts between them, and very few inter-marriages with each other, whereas we calculated an endogamy level ranging from 70% to 90%. Furthermore, various analyses of the Y chromosome, mitochondrial DNA and genome wide high density SNPs revealed a great deal of genetic differentiation among subpopulations within Ogliastra region [1 3].

***Study design, data collection and measurements***

Study design was cross-sectional and population-based. People living in the villages were invited to take part in the study by means of public proclamations and letters sent to every resident family. Respondents underwent blood sample tests, anthropometric, heart rate (HR) and blood pressure (BP) measurements. They were administered a standardized interview collecting socio demographic information, living habits, such as smoking (number of cigarettes smoked per day), alcohol consumption (number of glasses per day of wine, beer and spirits) and exercise (never/occasional/1-2 per week/ >2 per week), clinical and family history and medication history data.

BP was measured in both arms at initial assessment, at the end of a 40 minute interview and the arm with the higher pressure was used subsequently. After two or more measurements, performed according to the ESH guidelines [4], if the person had a systolic blood pressure (SBP) ≥ 140 mmHg or a diastolic blood pressure (DBP) ≥ 90 mmHg, he/she was advised to undergo a new measurement session over the next few weeks. BP measurements, as well as HR estimates, were obtained in the sitting position and with at least a two minute interval between each measurement. A standard mercury sphygmomanometer (Miniatur 300 B, Speidel & Keller) was used, and one of 3 cuff sizes (regular adult, large, or tight) was chosen on the basis of the circumference of the participant’s arm. BP measurements were made with the auscultatory method, Korotkoff phase I and phase V sounds being used in order to determine SBP and DBP levels. When phase V went to zero, phase IV was used. Hypertension or high BP was defined as mean clinic SBP≥ 140 mmHg, and/or mean clinic DBP≥ 90 mmHg or by the presence of ongoing treatment for hypertension, verifying the patient information slips the participants were invited to bring with them at the interview time. Awareness of hypertension was defined as the self-report of any prior diagnosis of hypertension made by a health care professional. Control of hypertension was defined as pharmacological treatment of hypertension associated with SBP<140 mmHg and DBP<90 mmHg.

Routine biochemical analyses and blood cell counts were all performed in our central laboratory in Perdasdefogu by a BT 3000 Targa Chemistry analyzer (Biotecnica Instruments, Rome, Italy) and a Coulter LH Hematology analyzer (Beckman-Coulter, Brea, CA).

Fieldwork, carried out by trained personnel, took place between 2002 and 2008. The research adheres to the tenets of the Declaration of Helsinki, furthermore written informed consent was obtained from all participants.

***Data analysis***

Statistical analyses were performed by using STATA 11 (StataCorp, College Station, TX). Descriptive statistics represent means, percentages, standard deviations and 95% confidence intervals (CI). One way ANOVA with Bonferroni correction was applied to examine differences in BP, anthropometric and serum parameters amongst Ogliastra villages adjusting for age and sex. Chi-square test, assessing frequencies of categorical variables, and t-test, assessing mean values of quantitative variables were used to compare men and women. Test on the equality of standard deviations (variances) in men and women was performed on SBP and DBP to evaluate differential variability by sex. Estimates of prevalence were standardized by the direct method to the age and sex structure of the 2008 Italian resident population. Linear regression was used to assess the relation between SBP and DBP, as continuous dependent variables, with the following independent variables: age, anthropometric measures, serum lipids, fasting glucose, uric acid, magnesium, sodium, potassium, calcium and white blood cells, separately by sex. Logistic regression was used to investigate the association between hypertension, as a dichotomous variable, with socio-economic and lifestyle variables we collected data on, and to evaluate the association of hypertension with its comorbidities, adjusting for the related covariates.

***Heritability analysis***

Thanks to the availability of complete municipal and parish archives going back to the seventeenth century, it was possible to recreate deep rooted genealogical trees (up to 16 generations) to connect all people living in the villages into large families with common ancestors. Using the software PedNavigator [5] the 9845 individuals in this study sample were included in 10 extended pedigrees, one for each village (mean size, 4530.5). We computed pair statistics running Pedstats [6]. Overall, the pedigrees count 45305 members, 6118 parent-offspring pairs, 7108 siblings, 1272 grandparent-grandchild pairs, 13949 avunculars, 151 half-siblings and 22070 first cousins. Although our quantitative genetic analysis only includes fully phenotyped individuals, it is best to use the largest possible pedigree for the computation of kinship matrices because genetic information is lost when pedigrees are broken up. In this kind of analysis it is typical that ancestors and founders are not phenotyped, but there is still great benefit from including them in the pedigree.

Since each village may represent a sub-isolate we performed separate heritability analyses in the ten villages on the following traits: SBP, DBP, SBP-DBP difference (pulse pressure), and HR. The heritability analysis was performed using a standard quantitative genetic variance-components model implemented in the software Sequential Oligogenic Linkage Analysis Routines (SOLAR ver 4.2.7) [7]. SOLAR is designed for incorporating all available information of extended pedigrees in parameters estimation. Heritability, the proportion of phenotypic variance attributable to additive genetic effects, was estimated for each BP measure after accounting for covariates whose effect was significant at the p<0.05 level. The significance of heritability estimates was tested by comparing the likelihoods of nested models using the likelihood ratio test.

To evaluate sex-specific effects on the variation of BP we used a standard sex-limitation modeling approach that allows testing for specific patterns of interaction, such as genotype by sex (G×S) interactions [8]. The expected genetic covariance between a male and female relative pair is defined as covariance (GM, GF)=2ϕ ρG(M,F) σGM σGF, where ϕ is the coefficient of kinship between the two individuals, ρG(M,F) the genetic correlation between the expressions of the trait in the two sexes, and σGM and σGF the genetic standard deviations (SD) for men and women. In the absence of a G×S interaction, the genetic correlation between relatives for a trait measured in males and females should be 1 and the genetic variances in the two groups should be equal. Conversely, if there is G×S interaction, the genetic correlation between the sexes, will be significantly <1 and/or the genetic variances will be different between the sexes. To evaluate G×S interactions influencing the traits, likelihood ratio test (LRT) was used to compare the 3 nested models, obtained constraining ρG=1, σGM=σGF and σEM=σEF respectively, to the full model, where σEM and σEF are the environmental SD in males and females.

In addition, we also carried out a bivariate quantitative genetic analysis to determine the extent of genetic and environmental covariation between systolic and diastolic BP and to investigate how much of the genetic component is shared between them and how much is trait specific. The phenotypic correlation between two traits is partitioned into additive genetic and random environmental components as given in the equation:

$$\rho_{P}=\rho_{G}\sqrt{h_{1}^{2}}\sqrt{h_{2}^{2}}+\rho_{E}\sqrt{1-h_{1}^{2}}\sqrt{1-h_{2}^{2}}$$

where ρ_G_ is the genetic correlation, ρ_E_ is the environmental correlation between trait pairs, and $h_{1}^{2}$ and $h_{2}^{2}$ are the heritabilities of trait 1 and trait 2, respectively [9]. Additive genetic correlations can range from −1 to 1, where a value of 1 indicates complete positive pleiotropy (the same genes are affecting the two traits in the same manner), a value of zero between the traits indicates that different genes influence them, and a value of −1 indicates complete negative pleiotropy (genes acting to increase the value of one trait decrease the value of the other one). A genetic correlation significantly different from both 0 and 1 (or −1) indicates incomplete pleiotropy, meaning that the two traits are influenced to some extent by the same genes or sets of genes, but that each trait also has a specific genetic basis. Finally, bivariate heritability, which is a measure of the extent to which shared genetic influences generate a correlation between two traits, is calculated as a function of the two univariate heritabilities and the genetic correlation [10].

**References**

1. Fraumene C, Petretto E, Angius A, Pirastu M (2003) Striking differentiation of sub-populations within a genetically homogeneous isolate (Ogliastra) in Sardinia as revealed by mtDNA analysis. Hum Genet 114: 1-10.
2. Fraumene C, Belle EM, Castrì L, Sanna S, Mancosu G, et al. (2006) High resolution analysis and phylogenetic network construction using complete mtDNA sequences in sardinian genetic isolates. Mol Biol Evol 23: 2101-2111.
3. Pistis G, Piras I, Pirastu N, Persico I, Sassu A, et al. (2009) High differentiation among eight villages in a secluded area of Sardinia revealed by genome-wide high density SNPs analysis. PLoS One 4: e4654.
4. 2003 European Society of Hypertension–European Society of Cardiology guidelines for the management of arterial hypertension. (2003) Journal of Hypertension 21: 1011–1053.
5. Mancosu G, Ledda G, Melis PM (2003) PedNavigator: a pedigree drawing servlet for large and inbred populations. Bioinformatics 19: 669-670.
6. Wigginton JE, Abecasis GR (2005) PEDSTATS: descriptive statistics, graphics and quality assessment for gene mapping data. Bioinformatics 21: 3445-3447.
7. Almasy L, Blangero J (1998) Multipoint quantitative-trait linkage analysis in general pedigrees. Am J Hum Gen 62: 1198-1211.
8. Martin LJ, Cole SA, Hixson JE, Mahaney MC, Czerwinski SA, et al. (2002) Genotype by smoking interaction for leptin levels in the San Antonio Family Heart Study. Genet Epidemiol 22: 105-115.
9. Lynch M, Walsh B. Genetics and analysis of quantitative traits. Sunderland, MA: Sinauer; 1998, p. 638.
10. Purcell S. Statistical methods in behavioural genetics. In: Plomin R, DeFries JC, McClearn, McGuffin P. Behavioural genetics. New York: Worth; 2008, p. 397.
